# Exposure to Indoor Mouldy Odour Increases the Risk of Asthma in Older Adults Living in Social Housing

**DOI:** 10.3390/ijerph16142600

**Published:** 2019-07-22

**Authors:** Loveth Moses, Karyn Morrissey, Richard A. Sharpe, Tim Taylor

**Affiliations:** 1 European Centre for Environment and Health, University of Exeter Medical School, Truro TR1 3HD, UK; 2 Public Health, Cornwall Council, Truro TR1 3AY, UK

**Keywords:** indoor environment, housing, mould, asthma

## Abstract

*Background:* Indoor dampness is thought to affect around 16% of European homes. It is generally accepted that increased exposure to indoor dampness and mould contamination (e.g., spores and hyphae) increases the risk of developing and/or exacerbating asthma. Around 30% of people in the Western world have an allergic disease (e.g., allergy, wheeze and asthma). The role of indoor mould contamination in the risk of allergic diseases in older adults is yet to be fully explored. This is of interest because older people spend more time indoors, as well as facing health issues due to the ageing process, and may be at greater risk of developing and/or exacerbating asthma as a result of indoor dampness. *Methods*: Face-to-face questionnaires were carried out with 302 participants residing in social housing properties located in South West England. Self-reported demographic, mould contamination (i.e., presence of mould growth and mouldy odour) and health information was linked with the asset management records (e.g., building type, age and levels of maintenance). Multivariate logistic regression was used to calculate the odd ratios and confidence intervals of developing and/or exacerbating asthma, wheeze and allergy with exposure to reported indoor mould contamination. We adjusted for a range of factors that may affect asthma outcomes, which include age, sex, current smoking, presence of pets, education, and building type and age. To assess the role of mould contamination in older adults, we compared younger adults to those aged over 50 years. *Results*: Doctor-diagnosed adult asthma was reported by 26% of respondents, 34% had current wheeze while 18% had allergies. Asthma was common among subjects exposed to reported visible mould (32%) and reported mouldy odour (42%). Exposure to visible mould growth and mouldy odour were risk factors for asthma, but not for wheeze or allergy. Exposure to mouldy odour increased the risk of asthma in adults over the age of 50 years (odds ratio (OR) 2.4, 95% confidence interval (CI) 1.10–5.34) and the risk was higher for females than for males (OR 3.5, 95% CI 1.37–9.08). These associations were modified by a range of built environment characteristics. *Conclusions*: We found that older adults living in social (public) housing properties, specifically women, may be at higher risk of asthma when exposed to mouldy odour, which has a number of implications for policy makers and practitioners working in the health and housing sector. Additional measures should be put in place to protect older people living in social housing against indoor damp and mould contamination.

## 1. Introduction

Asthma is a complex heterogeneous disease with variable severity, pathogenesis and treatment [1]. The nonspecific symptoms of asthma can be similar to symptoms of diseases such as chronic obstructive pulmonary disease (COPD) and can therefore make it difficult to estimate or diagnose in adults [2]. According to the World Health Organisation (WHO), about 235 million people worldwide are asthmatic and an estimated 383,000 people died in 2015 due to asthma [3]. The rapid rise in asthma and other allergic diseases (e.g., eczema and allergy) over the last several decades cannot be explained by genetic factors alone, which has led to an increased focus on environmental exposures. For example, asthma cases are expected to rise to 400 million globally by 2025 [1] due to increased exposure to air pollution, climate change, change in immune responsiveness, urbanisation and changes in lifestyle [1,4]. In the United Kingdom (UK), 4.3 million adults currently receive treatment for asthma [5]. It is thought that exposure to pollutants in the home may have a more pronounced impact on asthma because populations living in developed countries spend up to 90% of their time in the indoor environment [6], with approximately 69% spent in the residential indoor environment [7]. 

Significant progress has been made towards understanding the different factors in the indoor environment that can lead to the development and/or exacerbation of asthma and other allergic diseases. For example, exposure to combustion by-products such as those arising from environmental tobacco smoke, heating and cooking can contribute significantly to indoor exposure to diverse physical and chemical agents [8,9]. Exposure to indoor dampness and fungal contamination has been associated with a 30% to 50% increased risk of asthma [10,11,12,13]. Other predictors of mould contamination (e.g., presence of a mouldy odour) have been associated with a two-fold increased risk of asthma in an adult population [14]. With regard to fungal growth, common mould genera such as *Aspergillus, Alternaria, Candida, Cladosporium, Fusarium* and *Penicillium* that dominate in the indoor environment have been associated with asthma and other respiratory disorders [13,15,16]. Increased exposure to indoor dampness (e.g., rising damp, water penetration, leaks and condensation), and therefore mould, is a significant public health concern in Europe with around 16% of homes having problems with indoor dampness [17]. To date, much of the available evidence on the role of indoor damp and asthma concerns children [18,19,20,21] with much less work examining the relationship between indoor mould and asthma and other allergic disease in adulthood, particularly among older adult populations. This is despite exposure to indoor mould having a potentially higher risk for adults due to normal or pathological aging, and other risk factors such as underlying disease, socio-economic status (SES), diet and physical activity [22]. The limited research that has been conducted on adult populations found that exposure to mould contamination has been associated with doctor-diagnosed asthma, asthma symptoms, and bronchial hyperresponsiveness [23,24,25] and new onset asthma in adults [26]. Furthermore, this association has been found to be more pronounced for women, and especially in those with multi-sensitisation, and in particular mould sensitisation [26]. 

Within this context, this study investigates the level of indoor mould exposure and associated risk of asthma in older adults (i.e., over 50 years of age) living in social (public) housing in Cornwall, South West England. This is of interest because the impact of indoor dampness and related agents in older age is yet to be fully explored within a residential setting. This is relevant given current trends towards ageing societies whose members may spend more time indoors at home. Additionally, social housing in the UK generally supports older people and lower income households who experience greater problems with indoor dampness and mould contamination [14]. The remainder of the paper is structured as follows: Section 2 presents the methodology, including discussion of the design of the study and questionnaire; Section 3 presents the results of the statistical analysis; and Section 4 presents a discussion of the results and gives some conclusions. 

## 2. Methods and Materials

### 2.1. Study Background

This study forms part of the Smartline project, funded by the European Regional Development Fund. Smartline is a partnership project led by the University of Exeter, involving Coastline Housing (an independent, not-for-profit housing association), Cornwall Council and Volunteer Cornwall (a charity aiming to develop individuals and communities through voluntary action). Smartline has recruited over 300 households in properties owned and managed by Coastline Housing and has conducted a range of activities to better understand the health and wellbeing of individuals in these homes. Activities include the use of questionnaire surveys and qualitative interviews, and the collection of sensor data on indoor environments. Ethical approval for the Smartline project was granted by the University of Exeter.

### 2.2. Study Area/Population

**Figure 1:**
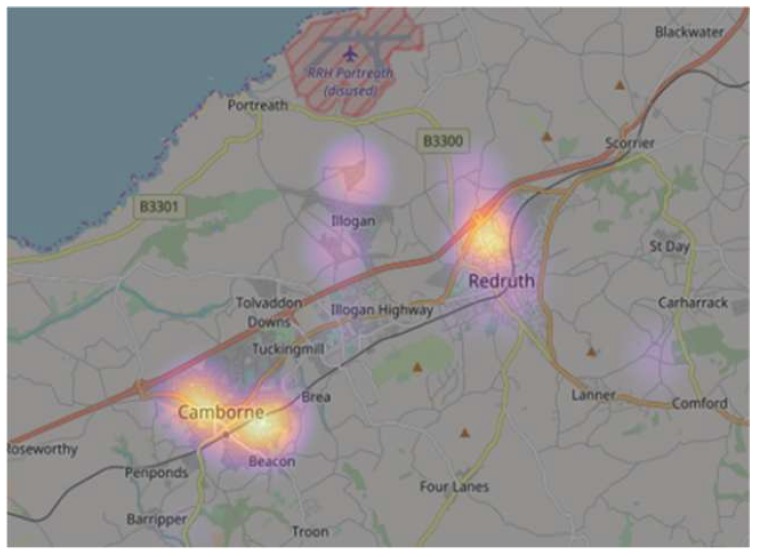
Map of Smartline sample residential locations.

This study focuses on a population residing in social housing in Cornwall, South West (SW) England. Cornwall has a strong maritime climate characterised by mild temperatures, strong winds and wet winters [14,27]. The area is largely rural with dispersed settlement patterns, and experiences high levels of deprivation [14,28]. The county is ranked 95 out of 326 local authorities in England for overall deprivation, and 121 out of 326 in terms of income deprivation in the 2015 Indices of Multiple Deprivation [29]. A quarter of our target population currently reside in neighbourhoods considered to be in the most deprived 20% in England [28]. The Smartline sample resides in properties owned and managed by Coastline Housing, a medium-sized not-for-profit social housing association. Coastline Housing owns and manages around 4000 homes spread out across West Cornwall (Figure 1). Our sample participants resided in the Camborne, Pool, Illogan and Redruth areas, and were all within a 10 square kilometer radius of each other forming one of Cornwall’s urban corridors. The close proximity of respondents ensured that broader environmental issues that may impact on both asthma and mould outcomes, such as climate and air pollution. were broadly consistent across the area of interest. 

In the UK, social housing associations are responsible for the provision of affordable housing to low-income populations [14]. In Cornwall, social housing associations are responsible for around 8% of the total housing stock [29], which represents approximately 12% of the population [30]. These homes are maintained according to UK Government’s Decent Homes Standards, 2006 [14,31], which means that properties are maintained and have slightly higher energy efficiency levels [14]. Due to the nature of the housing stock, the target population residing in social housing is generally more vulnerable with lower income households [14]; however, the age profile of this population is consistent across all age groups. This is in contrast to those that own their own home (who tend to be older adults) and those in the private rental sector (higher proportion of people aged from 16 to 34 years) [32].

### 2.3. Questionnaire Data

The questionnaire was designed using a closed questionnaire technique and was administered by trained enumerators and researchers over the period from January 2018 to June 2018, using questions on health and housing which broadly correspond with those used in a previous study on adults living in social housing in the area [14]. The six-month timeframe for data collection broadly encompassed winter, spring and early summer in the region. The overall survey lasted for approximately 45 minutes and included questions relating to demographic information, health and wellbeing, volunteering activity, use of technology, and data on the use of the home. In total, 311 households were surveyed. After discarding nine invalid/incomplete questionnaires, the sample size was reduced to 302 survey respondents. The elements of the survey relating to asthma risks were designed based on an earlier study on social housing [14] and collected information on all occupants, including demographic and environmental exposures thought to influence the risk of asthma. Questions included participant age, sex, height and weight; smoking status; employment; cleaning regimes; number of rooms carpeted; pets; health data on asthma, allergy and chronic bronchitis or emphysema; heating/ventilation regimes; and whether participants thought damp/mould impacted their family’s health [14]. Asthma in this study was defined as doctor-diagnosed asthma where participants confirmed they had seen a doctor and/or taken medication for asthma in the last 12 months. Wheeze was defined as adults who answered “yes” to having wheezing or dry coughing in the chest in the preceding 12 months. Allergy was defined as participants who answered “yes” to having an allergy and having seen a doctor in the last 12 months for allergy. The exposure variables for mould contamination were defined as the presence of visible mould (yes, no) and mouldy odour anywhere in the house (yes, no). Questionnaire data were merged with property records from Coastline Housing’s asset management and stock condition data (December 2018) using a unique household identifier. Data variables used in this study to account for the built environment included building age (pre-1930s, 1930–1965, 1965–1980s, 1980s onwards) and building type (house, bungalow, flat). 

### 2.4. Statistical Analysis

Descriptive analysis was used to describe the participants’ representativeness and differences in household demographics. The outcome data (asthma, wheeze and allergy) were analysed as separate dependent variables against the dichotomous mould exposures (reported visible mould, and mouldy odour). Univariate analyses were performed using logistic regression to assess the association between the variables of interest and asthma outcomes. Multivariate logistic regression analyses were used to estimate the relationship between reported mould exposures and asthma, wheeze, and allergy, adjusting for gender, age, smoking status, education, presence of pets, building type and building age. To evaluate the association between mould contamination and asthma across the life course, the asthma model was further stratified by age with a specific focus on adults aged 50 years and over. Associations are expressed as odds ratios (OR) with a 95% confidence interval (CI). All statistical analyses were performed using STATA/SE 15.0 (Stata Corporation, College Station, TX, USA).

## 3. Results

Table 1 presents descriptive statistics for the sample population. The average age of participants was 57 years (range 18–93 years). Sixty-nine percent of the survey participants were female and 39% were current smokers. Thirty-five percent of the participants were retired, while 27% had a long-term illness. Much of the population, 71%, had only completed formal primary and secondary (4–16 years) levels of education. Twenty-six percent of the adult participants had asthma and had seen a doctor for asthma in the last 12 months, while 34% reported having a wheeze in the preceding 12 months. Eighteen percent of adult participants reported having allergy in the preceding 12 months. Forty-five percent of the participants (47% female compared to 40% male) reported visible mould in different rooms in their homes. Eighteen percent of respondents reported having a mouldy odour in different rooms. Fifty-four percent of the participants lived in flats compared to 9% who lived in single-storey houses, and 35% of participants resided in properties less than 40 years old.

In order to assess the representativeness of our study population, we compared our sample to the demographic profile of those living in homes owned by Coastline. The demographic profile of our target population versus our study sample was similar in terms of mean age (59 years vs. 57 years, respectively); however, there was a higher proportion of women in our study sample compared to the target population (69% vs. 59%, respectively). In terms of the architectural type of the properties, a higher number of respondents lived in properties built after 1980 in our study sample compared to the target population (35% vs. 26%, respectively); and similarly for those living in flats (54% vs. 39%, respectively) [14].

The average age of those who reported having asthma and seeing a doctor in the last 12 months was 54.7 years, compared to 57 years for those who did not have asthma. Forty-three percent of those exposed to mouldy odour and 21% of those exposed to visible mould had current asthma. Reported asthma was more common among ex-smokers/current smokers and those in multiple occupancy housing. Asthma was also prevalent in those who had had wheeze or dry cough (55%), allergy (62%), and those who had seen a doctor for chronic bronchitis, emphysema or COPD (49%). 

### 3.1. Association between Behavioural and Indoor Exposure Covariates and Asthma

Younger adults (odds ratio (OR) 1.0, 95% confidence interval (CI) 1.00–1.10) and women (OR 2.7, 95% CI 1.4–5.1) were more likely to have asthma (Table 2). In terms of smoking, the risk of having doctor-diagnosed asthma was higher for current (OR 1.3, 95% CI 0.7–2.85) and ex-smokers (OR 1.4, 95% CI 0.6–2.80) compared to participants who never smoked, while there was a positive relationship with asthma for participants who smoked inside the home. However, these relationships were not statistically significant. There was also no clear relationship between vaping in the home and risk of asthma in this population. Those who vacuumed their house fewer than five times a month were more likely to have asthma than those who vacuumed more than five times a month. There were positive relationships between cat or dog ownership and asthma; however, the relationship was statistically stronger for dog ownership (OR 2.0, 95% CI 1.20–3.44) than for cat ownership (OR 1.6, 95% CI 0.92–2.73). Asthma was more common among those who had had wheeze or dry cough in the preceding 12 months (OR 14.55, 95% CI 1.83–116.05), had an allergy (OR 7.4, 95% CI 3.94–14.02), or had seen a doctor for chronic bronchitis, emphysema or chronic obstructive pulmonary disease (COPD) (OR 3.3, 95% CI 1.64–5.63). 

### 3.2. Exposure to Mould Contamination and Risk of Adult Asthma Diagnosis

There were no clear associations between self-reported visible mould growth and mouldy odour and the risk of asthma, wheeze and allergy (Table 3). The presence of visible mould growth may not be a true representation of the extent of contamination within a home, which may partly explain the relationship between the presence of a mouldy musty odour and asthma. In the unadjusted model, exposure to visible mould was not significantly associated with asthma (OR 1.4, 95% CI 0.86–2.42) but there was a significant association for reported mouldy odour (OR 2.7, 95% CI 1.46–5.02). After adjusting for our a priori covariates, exposure to a mouldy musty odour was associated with a 2-fold increased risk of asthma (Adjusted Odds Ratio (AOR) 2.7, 95% CI 1.32–5.58, *p* = 0.006) and of both allergy and asthma (AOR 2.2 ,95% CI 1.11–4.40, *p* = 0.025).

In our stratified adjusted model (Table 4), we found adults self-reporting the presence of a mouldy musty odour had a 4-fold increased risk of asthma (OR 4.0, 95% CI 1.4–11.14, *p* = 0.008). There was no association between mouldy musty odour and asthma among adults aged between 18 and 50 years. To assess the potential effects of demographic characteristics, we also stratified the model by gender. Compared to men, women had a 2-fold increased risk of asthma when exposed to mouldy odour (OR 2.4, 95% CI 1.02–5.67, *p* = 0.045).

## 4. Discussion

This study contributes to existing literature by investigating how exposure to mould contamination influences the risk of asthma in older adults. The study found that older adults were at greater risk of asthma when living in homes with a mouldy musty odour (i.e., a 4-fold increased risk of asthma in adults aged over 50 years). The study also demonstrates that women may be at greater risk, which may be due to gender differences in the amount of time spent indoors at home. Further research is needed to explore gender differences and possible variability in daily activities and time spent indoors. This will help improve future estimates of the impact of indoor dampness and related biological, chemical and physical air pollutants found in the home.

### 4.1. Synthesis with Existing Literature

It is generally accepted that indoor mould contamination increases the risk of asthma [10,11,13] across a range of populations. Other studies have reported significant associations between reported visible mould and the risk of adult asthma and allergy [14,24,25,26,33,34]. Prior research [14,33] supports our finding that exposure to a mouldy/musty odour has a stronger effect, with a 2-fold increased risk of adult asthma. The mechanism by which mouldy odours are associated with asthma may be related to toxic reactions in damp surfaces (e.g., resulting from the degradation of building materials), hypersensitivity reactions and degradation of microbial volatile compounds [35]. Also, the presence of a mouldy odour has been found to correspond with high a concentration of microbial contaminates and airborne mould concentration in homes [36]. Although a mouldy odour can be easily missed and not detected, this represents a strong indicator for poor air quality risk to adults with allergic and non-allergic disease [34]. The lack of association between self-reported visible mould growth and asthma and allergy in this study is discussed in full below. 

In terms of potential gender differences, studies have reported similar associations between mould contamination and risk of asthma in men and women [23]. However, in contrast to our findings, previous research also indicated that mould contamination had a stronger risk for asthma onset in younger adults [34]. While we were not able to formally test the mechanisms underpinning the reported gender and age results reported here, women and older adults may have increased exposures to indoor mould contamination due to different lifestyle characteristics and spending more time indoors at home. As described in Table 1, many of our participants were older and reported one or more chronic diseases, which in turn may make them less mobile and more likely to spend time indoors. At the same time, decreased mobility may impact on elderly residents’ capacity to clean/vacuum leading to increased mould growth in these households. Women, on the other hand, generally spend more time in the home, carrying out most of the house chores such as cleaning, caring and other activities making them more susceptible to mould exposure. For example, research [37] has found that frequent use of common household cleaning sprays may be an important risk factor for adult asthma. Moreover, late onset adult asthma is known to affect women particularly [38], which could also explain the findings of this study.

From a methodological perspective, the differences observed between this study and previous studies may be due to (i) the housing types and conditions of our sample population, and (ii) the behaviours in these lower income and more elderly households. With regard to housing conditions and type, our results may be influenced by the higher proportion of flats and new builds, and the previous retrofitting of homes in our study. Previous research has found that flats often have higher rates of severe mould contamination [39] and require greater ventilation rates in order to maintain humidity levels and avoid mould growth [35,40]. With regard to the age of the buildings, older properties are more likely to suffer from increased dampness from a lack of maintenance and degradation [35,41], and as such older properties have been associated with increased concentrations of moulds such as *Aspergillus* and *Penicillium* [42]. Research has also found that retrofitting homes with energy efficiency improvements (e.g., glazing, insulation, heating and draught-proofing may have a negative effect on health outcomes, with draught reduction linked to increased mould growth) [14]. Indeed, increased household energy efficiency in social housing properties has been found to increase the risk of adult asthma, which is likely to be a result of poor indoor air quality [14]. However, it is important to note that in this study, increased household energy efficiency was found to lower the risk of mould contamination in social housing [14]. 

With regard to residents behaviours, adequate ventilation is essential for the health and comfort of housing residents [43]. Mould contamination is influenced by heating and ventilation patterns within homes, which in turn may be linked to “fuel poverty” (the inability to adequately heat the home due to the cost of fuel). For example, research by Sharpe et al., in 2015 in the same target population, found that regardless of knowledge on the role of ventilation on health outcomes, people in fuel poverty are less likely to ventilate to save on heat and energy bills [44]. This is important to consider because the majority of properties managed by Coastline Housing rely on natural/passive ventilation to maintain air quality and moisture levels, and previous research has found that older generations see draughts as a “bad thing” and are less likely to use natural ventilation [45]. The presence of a mouldy odour is thought to be a strong marker for more severe mould contamination [14]. Therefore, although odour is dependent on occupants’ sense of smell it is a useful indicator for hidden (e.g., concealed behind ceilings, wallpaper and walls) mould contamination. As such, both visible mould growth and presence of a mouldy odour have been the focus of the majority of research in this area. While the presence of an odour has been consistently associated with the risk of developing and/or the exacerbation of asthma, there is a clear need for studies to utilise more novel complex molecular techniques [46]. The findings may also be a result of different outcome definitions between studies, particularly as this study did not assess the risk of asthma onset. 

With regard to the survey itself, the surveys were conducted over a six-month period, including winter, spring and summer months. The wide gap between survey commencement and completion, may explain our results, as previous research found that risk of asthma is higher in winter months, whilst warmer/drier summer months can influence mould growth within the home [47]. Future studies need to consider the possible impact of time and seasonality [48] on mould growth and asthma outcomes in their research design and sample size calculations. Mould contamination and resultant health effects may also be influenced by varying climatic conditions across SW England. Cornwall experiences a wetter and milder climate than the rest of the UK [27,49], which can provide ideal conditions for mould growth, particularly in the winter months [50]. 

However, it is important to note that our topic of interest is complex and is the result of complex interactions between residents’ lifestyles and the natural and built environment, which includes both housing conditions and also external environmental conditions, such as outdoor air pollution and access to greenspace. It is also important to consider the impact of other allergens found in the home and their interactions with occupant health outcomes. For example, it is thought that the diversity of indoor allergens and allergic sensitisation may play a different role in children and adults [34], which further highlights the complexity of investigating the impact of allergic diseases in older age. It is also likely that the association between COPD and asthma may partly explain our findings. 

### 4.2. Strengths and Limitations

A strength of this study was its response rate. After discarding nine invalid/incomplete questionnaires, a comparatively high proportion of the targeted population (87%) participated in the study. This reduced the risk of bias within this population because the potential of selection bias is reduced in response rates above 62% [51]. The questionnaires were completed by trained enumerators to ensure questions were explained to participants and to reduce the risk of information bias through misunderstanding/misinterpretation of the questions. This also may have led to participants being more engaged, hence the increase in study response. The focus on social housing residents means that the project provides a unique insight to this sub-population. A further strength of this study is its innovative focus on the elderly population. As noted, to date the evidence base on asthma has focused on children and young adults. How this paper indicates that future work needs to also focus on older populations, particularly those living in more deprived areas. 

However, some limitations do exist. The cross-sectional study design limits the possibility of drawing conclusions on causality, and the sample size reduces our confidence in the effect sizes (resulting in wide confidence intervals). There is the potential for bias resulting from participants believing that indoor damp and/or mould contamination impacts their family’s health [14], and the potential for asthmatics being more likely to report dampness related issues [10]. As indicated above, it is also possible that a lack of heating and ventilation, and elevated dampness, may lead to the proliferation of house dust mites and concentrations of volatile organic compounds which are known risk factors for asthma [35]. Our self-report exposure outcomes may also introduce an element of uncertainty, but it is clear that homes with visible mould growth have higher concentrations of microbial exposures [36]. While the use of self-reported measures may introduce an element of error, others have found that self-reported asthma has corresponded well with a general practitioner (GP) formal diagnosis [52]. The prevalence of asthma in our study was high (26%) when compared to the UK general population; however, similar prevalence rates have been reported by prior studies assessing the risk of asthma in lower income populations [14,53].

Studies that compared self-reported mould exposures with site visits and/or quantitatively measured microbial exposures have also shown that occupants may underestimate their exposures [54], or may not suspect or detect mould contamination [36]. Our findings may also have been influenced by other building characteristics not assessed in this study, which may include building type, ventilation type and indoor temperature, etc. These factors modify indoor mould concentration and/or risk of asthma. To further our understanding of the impact of indoor environmental exposures, future interdisciplinary studies must account for these limitations. This could be achieved through larger scale studies investigating health risks associated with indoor air pollution through the use of air pollution sensors and more detailed information on behavioural and building characteristics.

## 5. Conclusions

Exposure to indoor mould contamination in social housing increases the risk of doctor-diagnosed asthma, which appears to be greatest among older adults and women. It is important to note that our topic of interest is complex and is the result of complex interactions between residents’ lifestyles and the natural and built environment, which includes both housing conditions and also external environmental conditions, such as outdoor air pollution and access to greenspace. However, this study indicates that there is a clear need for housing providers to improve housing conditions in social housing communities, as well as improve occupants’ awareness of indoor mould effects and educate them on strategies to reduce mould contamination to help avoid these adverse health outcomes. Furthermore, the complex interactions and feedback loops between resident behaviours, housing type and environmental conditions, as documented in the discussion, mean that future planning needs a more holistic and “whole house” approach that accounts for resident behaviours and the built environment, rather than the often piecemeal approach to housing that dominates the current policy landscape [7]. The findings from this study can be applied to other comparable low-income populations; however, it is vital to consider the possible bias in the study when discussing the results or applying the findings to the general population. 

## Figures and Tables

**Table 1:** Descriptive statistics of Smartline participants.

Socio-Demographic Characteristics	Study Participants	
	Percentage (%)	Number (*n* = 302)
Percentage male	30	91
Percentage female	69	208
Mean adult age (≥18 years) = 57, range = 18–93 years		
Smoking status		
Non-smoker	24	72/302
Ex-smoker	37	113/302
Current smoker	39	116/302
Smoke <5 times a day	15	44/302
Smoke 5–15 times a day	34	102/302
Smoke more than 15 times a day	27	81/302
Smoke inside house	34	101/299
Respiratory Health		
Adults with a wheeze or dry cough in ≤12 months	34	104/302
Have asthma and seen a doctor in ≤12 months, for asthma	26	78/302
Allergy	18	55/302
Emphysema or chronic bronchitis	13	39/302
Education		
GCSE/O-level or lower (including no formal qualification)	71	212/302
A-level or equivalent	25	74/302
Degree level or equivalent	4	13/302
Participants providing employment status		
Employed	18	55/302
Unemployed	0	0
Actively looking for work	2	7/302
Retired	35	107/302
Long-term illness	27	82/302
Housekeeper/carer	14	42/302
Student	2	5/302
Other	1	4/302
Presence of pets in the house		
Cat	31	93/302
Dog	36	110/302
Reported visible mould contamination	45	135/302
Reported mouldy odour	18	54/292
Household occupancy (mean = 2, range 1–7)		302
1-single occupancy	42	128/302
2-double occupancy	31	93/302
3-triple occupancy	13	40/302
4+ multiple occupancy	14	41/302
Build age of properties (1929–2016, mean = 45 years)		302
Pre-1930	3	9
1930–1965	41	124
1965–1980	20.5	62
1980+	35	107
Build Type		273
House	37	101
Bungalow (single-storey house)	9	24
Flat (apartment)	54	148

**Table 2:** Univariate analyses between covariates and asthma.

Covariates	Current Asthma	
	% (*n*/d)	OR (95% CI)
**Participant Age (Mean = 54.7)**		
≤50 years	32 (33/103)	1.0 (1.00–1.10)
>50 years	23 (45/197)	0.98 (0.95–1.01)
Gender		
Male	14 (13/91)	Ref
Female	31 (64/208)	2.7 (1.4–5.14) **
Smoking history		
Never smoked (ref)	22 (16/72)	Ref
Ex-smoker	28 (31/112)	1.4 (0.69–2.80
Current smoker	28 (31/113)	1.3 (0.70–2.85)
Smoke inside house		
No	25 (49/198)	Ref
Yes	28 (28/100)	1.2 (0.68–2.04)
Home Vape		
No	26 (66/254)	Ref
Yes	26 (11/43)	0.98 (0.47–2.05)
Vacuum house (n per month quartiles)		
0–5 times	29 (22/77)	1.2 (0.42–3.21)
5–15 times	23 (21/92)	0.89 (0.31–2.52)
15–30 times	28 (29/104)	1.2 (0.42–3.21)
>30 (ref)	25 (6/24)	Ref
Household occupancy		
1-Single occupancy	23 (30/128)	Ref
2-double occupancy	23 (21/93)	0.95 (0.50–1.80)
3-triple occupancy	35 (14/40)	1.8 (0.82–3.79)
Multiple occupancy (4+)	33 (13/40)	1.5 (0.72–3.42)
Wheeze or dry cough in ≤12 months		
No	8 (1/13)	Ref
Yes	55 (57/104)	14.55 (1.83–116.05) *
Allergy		
No	18 (44/246)	Ref
Yes	62 (34/55)	7.4 (3.94–14.02) ***
Seen a doctor for chronic bronchitis, emphysema or COPD		
No	23 (59/262)	Ref
Yes	49 (19/39)	3.3 (1.64–6.53) **
Presence of Cat		
No	23 (48/208)	Ref
Yes	36 (30/93)	1.6 (0.92–2.73)
Presence of Dog		
No	21 (40/192)	Ref
Yes	35 (38/109)	2 (1.20–3.44) **
Home has more than 2 rooms carpeted or rugged		
No	30 (28/93)	Ref
Yes	35 (50/208)	0.7 (0.43–1.27)
Build age		
Pre-1930	29 (2/7)	1.3 (0.25–6.88)
1930–1965	52 (42/81)	2.4 (1.29–4.47)
1965–1980	32 (15/47)	1.5 (0.69–3.17
1980+	22 (19/88)	Ref
Build Type		
House	33 (6/18)	Ref
Bungalow	43 (30/70)	1.3 (0.46–3.56)
Flat (apartment)	33 (37/111)	1 (0.37–2.707)

Note: Ref: reference category; * *p* < 0.05; ** *p* < 0.01; *** *p* < 0.001.

**Table 3:** Association between indoor mould contamination and risk of adult asthma, wheeze and allergy.

Health Outcome	Presence of Visible Mould		Presence of Mouldy Odour	
	Unadjusted	Adjusted	Unadjusted	Adjusted
	OR (95% CI)	OR (95% CI)	OR (95% CI)	OR (95% CI)
Current Asthma	1.4 (0.86–2.42)	1.02 (0.55–1.89)	2.7 (1.46–5.02) **	2.7 (1.32–5.58) **
Wheeze	1.3 (0.41–4.18)	1.2 (0.28–4.91)	0.7 (0.20–2.62)	0.93 (0.18–4.81)
Allergy	0.98 (0.54–1.76)	1.04 (0.52–2.09)	0.97 (0.45–2.07)	0.95 (0.40–2.30)
Current Asthma and Allergy	1.2 (0.77–2.02)	1.0 (0.6–1.84)	2.3 (1.26–4.18) **	2.2 (1.1–4.40) *

Note: Ref: reference category: * *p* < 0.05; ** *p* < 0.01.

**Table 4:** Indoor mould and risk of asthma in adults aged over 50 years.

Mould Contamination	Health Outcome: Adult Asthma				
	Percent (*n*/d)	Unadjusted		Adjusted	
		OR 95% CI		OR 95% CI	
Presence of visible mould					
≤50 years	33 (22/62)	Ref		Ref	
>50 years	31 (19/72)	1.3	0.65–2.53	1.02	0.50–2.30
Presence of mouldy odour					
≤50 years	100 (13/27)	Ref		Ref	
>50 years	38 (11/27)	2.6	1.10–6.14 *	4.0	1.40–11.14 **

Note: Ref: reference category: * *p* < 0.05; ** *p* < 0.01.
